# Association of the Salivary Progesterone-to-Estradiol Ratio with Threatened Miscarriage

**DOI:** 10.3390/jcm15103703

**Published:** 2026-05-12

**Authors:** Hande Kurt Güven, Mehmet Efe Namlı, Çağanay Soysal, Sedat Özdemir, Eda Arzu Şahin, Elif Yılmaz

**Affiliations:** 1 Department of Obstetrics and Gynecology, Ankara Atatürk Sanatorium Research and Training Hospital, Ankara 06010, Turkey; drmefenamli@gmail.com (M.E.N.);; 2 Department of Obstetrics and Gynecology, Ankara Etlik City Hospital, Ankara 06170, Turkey; cagnay@hotmail.com; 3 Clinics of Biochemistry, Ankara Atatürk Sanatorium Research and Training Hospital, Ankara 06170, Turkey

**Keywords:** threatened miscarriage, salivary hormones, progesterone, estradiol, progesterone/estradiol ratio

## Abstract

**Objective:** To evaluate the association of salivary progesterone and estradiol levels and the progesterone-to-estradiol ratio (P4/E2 ratio) in early pregnancy with threatened miscarriage and pregnancy outcome. **Methods:** This single-center prospective cohort study was conducted at the Department of Obstetrics and Gynecology, Ankara Atatürk Sanatorium Training and Research Hospital, between 10 January 2025 and 10 January 2026. Primigravid women with a confirmed live intrauterine pregnancy were included. Saliva samples were collected at 6–7 and 8–9 weeks of gestation; in pregnancies remaining viable to 12 weeks, salivary estradiol was also measured at week 12. Progesterone and estradiol levels were measured by immunoassay, and the P4/E2 ratio was calculated. Participants were classified as healthy ongoing pregnancies to 12 weeks (*n* = 102), threatened miscarriage with viability preserved to 12 weeks after progesterone treatment (*n* = 63), and miscarriage (*n* = 29). Group comparisons were performed using the Kruskal–Wallis test, one-way ANOVA, Mann–Whitney U test, and paired-samples *t*-test, as appropriate. **Results:** At both 6–7 and 8–9 weeks, progesterone, estradiol, and the P4/E2 ratio differed significantly among groups. Values were highest in uncomplicated ongoing pregnancies, lowest in pregnancies ending in miscarriage, and generally intermediate in progesterone-treated threatened miscarriage with preserved viability. The progesterone-to-estradiol ratio increased significantly from 6–7 to 8–9 weeks in uncomplicated ongoing pregnancies (*p* = 0.005), whereas no significant longitudinal change was observed in the threatened miscarriage group. Among pregnancies viable at 12 weeks, estradiol levels at week 12 remained significantly lower in women with prior vaginal bleeding (*p* < 0.001). **Conclusions:** Salivary progesterone, estradiol, and the progesterone-to-estradiol ratio were significantly associated with early pregnancy outcome in this prospective cohort. These findings suggest that salivary hormonal assessment may provide a non-invasive adjunct for characterizing early hormonal patterns in threatened miscarriage; however, it should not be interpreted as a stand-alone prognostic tool without further validation in larger cohorts.

## 1. Introduction

Threatened miscarriage is one of the most common obstetric problems encountered in early pregnancy and is characterized by vaginal bleeding occurring in the presence of a live intrauterine pregnancy without cervical dilatation [1]. The clinical course is not the same in every patient; while some pregnancies continue uneventfully, in some cases a more adverse process associated with pregnancy loss or later obstetric complications may be observed [2]. This heterogeneous picture often renders evaluation based solely on clinical symptoms insufficient and increases interest in easily applicable biological markers that may better reflect prognosis [1,3].

The continuation of early pregnancy depends not only on individual hormone levels but also on the delicate balance between estradiol and progesterone [4,5]. While estradiol supports endometrial proliferation and the formation of an appropriate uterine environment capable of responding to progesterone, progesterone has a more decisive role in maintaining decidualization, preserving the endometrial structure favorable for implantation, and shaping immune tolerance at the maternal-fetal interface [4,6]. Therefore, in clinical conditions such as threatened miscarriage, where the biological fragility of pregnancy is at the forefront, evaluating these two hormones together may be a more meaningful approach than considering a single hormone level alone [4]. Indeed, recent studies have shown that serum progesterone measurement may have a certain prognostic value in predicting pregnancy loss in women with threatened miscarriage and that estradiol is also among the biochemical markers associated with early pregnancy loss [3,7].

Measurement of steroid hormones in saliva has attracted increasing interest in recent years because it is less invasive than blood sampling and offers an approach more suitable for repeated sampling [8]. The ability of steroid concentrations in saliva to reflect the biologically active fraction not bound to circulating carrier proteins also makes this type of sampling conceptually strong in studies aiming to evaluate hormonal balance [8]. Indeed, recent data extending from pregnancy to the postpartum period have revealed that estradiol and progesterone can be monitored in saliva and that the levels of these hormones show significant variability throughout pregnancy [9].

In recent years, studies aimed at predicting pregnancy outcome in women with threatened miscarriage have focused on the prognostic value of serum progesterone, models derived from routine blood tests, and risk classification tools combining clinical-radiological variables with biochemical parameters [1,3]. However, an important part of this literature proceeds through venous blood samples and ultrasound findings, whereas non-invasive hormonal sampling approaches remain relatively in the background [3,8]. Although recent analytical studies have shown that sex steroids in saliva are measurable and can be reliably monitored with appropriate methods, data clarifying the clinical relevance of these measurements in early pregnancy complications such as threatened miscarriage are still limited [8,9]. In particular, although a ratio-based salivary evaluation in which estradiol and progesterone are considered together has the potential to provide a more informative picture than individual hormone levels, it has not been sufficiently defined in the current literature in the context of threatened miscarriage [3,9].

Therefore, although the current data obtained through serum progesterone levels and clinical risk models provide useful clues in women with threatened miscarriage, the place of non-invasive hormonal sampling in this clinical picture has still not been sufficiently clarified [1,3]. Although it has been shown that sex steroids in saliva are analytically measurable, data examining the relationship between a ratio-based approach in which estradiol and progesterone are evaluated together and threatened miscarriage are quite limited [8,10]. We hypothesized that women with threatened miscarriage, particularly those who subsequently experienced pregnancy loss, would have lower salivary progesterone and estradiol levels and a lower progesterone-to-estradiol ratio than women with uncomplicated ongoing pregnancies. Therefore, in this study, we aimed to investigate the relationship between salivary progesterone-to-estradiol ratio and threatened miscarriage and to evaluate whether this ratio and the related hormonal parameters differ between pregnant women with threatened miscarriage and healthy pregnant controls.

## 2. Materials and Methods

This single-center, prospective cohort study was conducted at the Department of Obstetrics and Gynecology, Ankara Atatürk Sanatorium Training and Research Hospital, between 10 January 2025 and 10 January 2026. Primigravid pregnant women with confirmed viability at 6–7 weeks of gestation were included in the study. At the baseline assessment, saliva samples were obtained from all participants, and estradiol (E2) and progesterone (P4) levels were measured. The progesterone-to-estradiol ratio (P4/E2 ratio) was calculated by dividing salivary progesterone concentration by salivary estradiol concentration. The main aim of the study was to compare salivary estradiol, progesterone, and progesterone-to-estradiol ratios among the three clinical outcome groups at baseline and during follow-up, and to evaluate whether these parameters were associated with first-trimester pregnancy outcome.

The inclusion criteria for the study were defined as confirmed live intrauterine pregnancy at 6–7 weeks of gestation, presence of a primigravid pregnancy, and provision of written informed consent for participation in the study. The exclusion criteria were defined as pregnancies over 12 weeks, presence of bleeding diathesis, history of recurrent miscarriage, regular medication use, presence of vaginal bleeding due to causes other than threatened miscarriage (cervical polyp, cervical erosion, etc.), chronic diseases (diabetes, hypothyroidism or hyperthyroidism, etc.), non-completion of the follow-up process, and refusal to participate in the study.

Salivary E2 and P4 measurements were performed in all participants at 6–7 weeks of gestation. A second saliva sample was obtained from the same participants at 8–9 weeks of gestation. In women presenting with vaginal bleeding, vaginal progesterone treatment was initiated as part of routine clinical care at the time of clinical diagnosis of threatened miscarriage. The treatment consisted of vaginal progesterone at a dose of 200 mg/day. In women with threatened miscarriage, saliva samples were collected before the first dose of progesterone. Patients who could not be reached at 8–9 weeks of gestation were excluded from the study. Pregnant women were followed until the 12th gestational week and categorized into three clinical outcome groups. Pregnancies that remained viable up to the 12th week without vaginal bleeding were classified as the uncomplicated ongoing pregnancy group (*n* = 102). Women who presented with vaginal bleeding, received progesterone treatment as part of routine clinical care, and remained viable up to the 12th week were classified as the progesterone-treated threatened miscarriage with preserved viability group (*n* = 63). Women who presented with vaginal bleeding, received progesterone treatment, but subsequently experienced pregnancy loss before the 12th week were classified as the progesterone-treated threatened miscarriage ending in pregnancy loss group (*n* = 29). At the end of the study, the total number of participants was 194. Progesterone treatment was not assigned as a study intervention but was initiated according to routine clinical practice in women presenting with vaginal bleeding. Therefore, the threatened miscarriage groups in this study represent treated clinical subgroups rather than untreated natural-history groups.

Saliva samples were collected from the participants at three different time points: at 6–7 weeks, salivary E2 and P4 measurements were performed in all participants; at 8–9 weeks, saliva samples were again obtained from all participants and E2 and P4 levels were re-evaluated; at 12 weeks, salivary E2 measurement was performed only in pregnancies that remained viable up to the 12th week, that is, in those who had bleeding and received progesterone treatment and in pregnant women without bleeding. Thus, it was aimed to compare hormonal changes in early pregnancy on a group basis.

Saliva samples were obtained using the passive collection technique. All participants were asked not to eat, drink, or perform oral care for 30 min before sampling. At the end of this period, saliva samples were collected into sterile tubes and stored at −80 °C until the day of analysis. Before analysis, the samples were thawed at room temperature and then centrifuged at 4000 rpm for 20 min. Before sample analysis, the analyzer (Abbott Alinity i device (Abbott Laboratories, Chicago, IL, USA)) was calibrated according to the manufacturer’s recommendations, and internal quality-control procedures were performed by the biochemistry laboratory. All salivary hormone measurements were performed using the same analyzer under standardized laboratory conditions. Hormone concentrations were reported in pg/mL according to the output of the immunoassay platform used by the biochemistry laboratory. Before analysis, the analyzer was calibrated according to the manufacturer’s recommendations, and internal quality-control procedures were performed as part of the routine laboratory workflow. The same analytical platform, reagent system, calibration procedure, and quality-control process were used for all samples to minimize analytical variability. Hormone concentrations were reported quantitatively in pg/mL according to the assay output, and the reported numerical values were used for statistical analysis without additional transformation. Salivary measurements were interpreted within the context of this standardized study protocol and were not considered directly interchangeable with serum-based clinical hormone measurements.

Ethical approval for this study was obtained from the Ethics Committee of Ankara Atatürk Sanatorium Training and Research Hospital (Approval No: E-53610172–799-265027705, Date: 10 January 2025). The study was conducted in accordance with the principles of the Declaration of Helsinki. Written informed consent was obtained from all participants.

### Statistical Analysis

Statistical analyses were performed using IBM SPSS Statistics version 27. Descriptive statistics were presented as mean ± standard deviation for normally distributed continuous variables, median [interquartile range] for non-normally distributed continuous variables, and number and percentage for categorical variables. The distributional characteristics of continuous variables were evaluated before selecting parametric or non-parametric tests. For comparisons among three independent groups, one-way ANOVA was used for variables compatible with normal distribution, and the Kruskal–Wallis H test was used for variables not compatible with normal distribution. Post hoc comparisons were performed using Bonferroni correction for non-parametric comparisons and Tamhane’s T2 test when the assumption of homogeneity of variance was not met. For comparisons between two independent groups, the independent-samples *t*-test or Mann–Whitney U test was used, as appropriate. For paired within-group comparisons between 6–7 and 8–9 weeks, the paired-samples *t*-test was used for normally distributed variables. Categorical variables were compared using Pearson’s chi-square test. A *p* value of <0.05 was considered statistically significant.

## 3. Results

Baseline characteristics are presented in Table 1. Maternal age differed significantly among the groups (χ^2^ = 25.760, *p* < 0.001), with the progesterone-treated threatened miscarriage with preserved viability group being older than both the uncomplicated ongoing pregnancy and miscarriage groups. BMI also differed significantly among the groups (χ^2^ = 8.400, *p* = 0.015), with lower values observed in the miscarriage group than in the other two groups. Employment status, education level, and income level did not differ significantly among the groups (all *p* > 0.05) (Table 1).

At 6–7 weeks of gestation, salivary progesterone, estradiol, and the progesterone-to-estradiol ratio differed significantly among the three groups (all *p* < 0.001). The highest values were observed in the uncomplicated ongoing pregnancy group, whereas the lowest values were observed in pregnancies ending in miscarriage. The progesterone-treated threatened miscarriage with preserved viability group generally showed intermediate progesterone and estradiol concentrations. Pairwise comparisons showed that the uncomplicated ongoing pregnancy group differed significantly from both threatened miscarriage groups for all three hormonal parameters. In addition, progesterone and estradiol levels were significantly higher in the progesterone-treated threatened miscarriage with preserved viability group than in the miscarriage group. A similar pattern was observed at 8–9 weeks. Salivary progesterone, estradiol, and the progesterone-to-estradiol ratio again differed significantly among the three groups (all *p* < 0.001). Values remained highest in uncomplicated ongoing pregnancies and lowest in pregnancies ending in miscarriage. Pairwise comparisons showed significant differences between uncomplicated ongoing pregnancies and both threatened miscarriage groups for all hormonal parameters, while progesterone and estradiol levels also differed significantly between the two threatened miscarriage subgroups. For the progesterone-to-estradiol ratio, post hoc comparisons showed significantly higher values in the uncomplicated ongoing pregnancy group than in both threatened miscarriage subgroups, whereas the two threatened miscarriage subgroups did not differ significantly from each other. It was determined that the 8–9 week progesterone/estradiol ratio values of those who were healthy pregnant up to the 12th week were significantly higher than those with threatened miscarriage and those who had miscarriage (Table 2).

The progesterone-to-estradiol ratio increased significantly from 6–7 to 8–9 weeks in the uncomplicated ongoing pregnancy group (t = −2.901, *p* = 0.005). In contrast, no significant longitudinal change was observed in the progesterone-treated threatened miscarriage with preserved viability group (*p* > 0.05) (Table 3).

A statistically significant difference was detected among the groups in terms of 6–7 week estradiol values (Z = −3.920; *p* < 0.001). It was determined that the 6–7 week estradiol values of those who had uncomplicated ongoing pregnancies were significantly higher than those with threatened miscarriage.

A statistically significant difference was detected among the groups in terms of 12th week estradiol values (Z = −9.025; *p* < 0.001). It was determined that the 12th week estradiol values of those who had uncomplicated ongoing pregnancies were significantly higher than those with threatened miscarriage (Table 4).

## 4. Discussion

The main finding of this study is that salivary progesterone and estradiol levels were higher at both 6–7 and 8–9 weeks of gestation in pregnancies that continued healthily up to the 12th gestational week. Pregnancies with threatened miscarriage do not appear to be hormonally either completely normal or completely compatible with miscarriage; most of the time, they exhibit a profile between these two conditions. Our findings are consistent with recent studies reporting an association between lower progesterone levels and pregnancy loss in women with threatened miscarriage [1,3]. Similarly, the fact that lower or insufficiently increasing estradiol levels in early pregnancy have been found to be associated with adverse pregnancy outcomes supports that the estradiol differences observed in our study may be biologically meaningful [7,11]. However, these findings should be interpreted as group-level associations rather than evidence of independent prognostic performance or causal hormonal mechanisms.

Our findings suggest that a favorable outcome in early pregnancy may be associated not only with higher progesterone and estradiol levels, but also with a higher progesterone-to-estradiol ratio. In our study, the higher progesterone-to-estradiol ratio in uncomplicated pregnancies suggests an association between this ratio and early pregnancy viability. Progesterone not only supports endometrial receptivity and decidualization. Studies also show that progesterone plays a role in maintaining immune tolerance in the maternal-fetal relationship [4,6]. Estradiol is also an important hormone in early pregnancy in terms of endometrial preparation and uteroplacental adaptation. However, rather than being high or low on its own, preservation of the physiological balance it establishes with progesterone appears to be more meaningful [4,7]. This interpretation should be limited to association because the present observational design does not allow causal inference. Because the current data alone do not reveal whether hormonal imbalance is the direct cause of pregnancy loss or a concomitant biological reflection of it [3].

In our study, although the progesterone/estradiol ratio increased significantly from 6–7 weeks of gestation to 8–9 weeks in the control group, a similar change was not observed in the progesterone-treated threatened miscarriage with preserved viability group. This suggests that in early pregnancy, not only a single hormonal measurement, but also the hormonal course itself may be biologically important. It is known that sex steroids physiologically increase throughout pregnancy in healthy pregnancies and that this increase reflects an adaptive endocrine response accompanying the maintenance of pregnancy [9]. In addition, one study showed that a regularly increasing estradiol curve in early pregnancy was associated with a lower risk of early pregnancy loss, thus revealing that dynamic hormonal patterns may carry more information than single-time measurements [11]. Similarly, the finding that repeated decreases in serum progesterone are associated with early pregnancy loss supports that disruption in progesterone dynamics may also be linked to adverse pregnancy outcomes [12]. Within this framework, our findings suggest that the temporal pattern of the progesterone-to-estradiol ratio may differ between uncomplicated pregnancies and pregnancies accompanied by early vaginal bleeding.

In our study, when the cases that remained viable up to the 12th gestational week were examined within themselves, salivary estradiol levels were lower in the group that had previously received progesterone treatment because of vaginal bleeding than in pregnancies with an uneventful course. This suggests that the hormonal divergence that emerges in the early period is not only a transient change accompanying the acute bleeding period. Reviews showing that sex steroids physiologically increase from early pregnancy to the postpartum period demonstrate that an increase in estradiol is an expected condition during the healthy continuation of pregnancy [9]. In addition, it has been reported that high and regularly increasing estradiol levels in the early pregnancy period in naturally conceived pregnancies are associated with a lower risk of pregnancy loss. This supports the possibility that estradiol dynamics may reflect biological differences associated with early pregnancy viability [11,13]. Therefore, our findings suggest that in pregnancies that remain viable up to the 12th week but are accompanied by bleeding in the early period, the hormonal profile does not completely “normalize” and that these cases may constitute a more endocrinologically fragile subgroup. In addition, meta-analytic data reporting that first-trimester vaginal bleeding is associated with more adverse obstetric outcomes show that this interpretation is also clinically important [2]. However, the clinical meaning of lower estradiol levels at 12 weeks in women with prior bleeding remains uncertain and should be considered hypothesis-generating.

A potentially relevant aspect of these findings is that saliva-based hormonal assessment is non-invasive, repeatable, and more acceptable for serial sampling than venous blood collection. A study on steroid hormone measurement in saliva showed that, with appropriate methods, saliva samples can reflect the biologically active free hormone fraction and provide a practical basis for clinical studies requiring serial sampling [8]. On the other hand, considering that current risk classification models developed for threatened miscarriage still mostly rely on clinical, biochemical, and ultrasonographic variables, salivary progesterone, estradiol, and their ratio may serve as complementary markers to clinical and ultrasonographic assessment, rather than as stand-alone diagnostic or prognostic tests [1,3]. However, before clinical implementation, clinically meaningful cut-off values, standardized salivary assay protocols, multivariable predictive models, and external validation in independent cohorts are required.

The main strengths of our study are that it has a prospective cohort design, that salivary sampling was performed at multiple time points in the same pregnancy, and that both threatened miscarriage cases and cases resulting in miscarriage were evaluated together with pregnancies with a healthy course. In these respects, the study is consistent with the current approach emphasizing that the risk of early pregnancy loss should be understood not only on the basis of a single clinical observation but also on biological change over time [1].

However, the study has some important limitations. One of these is that it was conducted at a single center and that the number of cases in the miscarriage group remained lower than in the other groups. The exclusion of women with recurrent miscarriage, chronic systemic disease, or regular medication use may limit the generalizability of the findings to the broader threatened miscarriage population encountered in routine clinical practice. In addition, maternal age and BMI differed significantly among the groups, and these differences may have acted as potential confounders in the association between salivary hormonal parameters and pregnancy outcome. Because multivariable adjustment was not performed, residual confounding related to age, BMI, or other unmeasured factors cannot be excluded. Because progesterone treatment was initiated in women presenting with vaginal bleeding as part of routine clinical care, the threatened miscarriage groups represent treated clinical subgroups rather than untreated natural-history cohorts. Therefore, although our findings are promising, they should be supported by studies with larger sample sizes and external validation. Another methodological limitation is related to the use of saliva as the sample matrix for immunoassay-based hormone measurement. Although saliva sampling provides a non-invasive and repeatable approach, specific manufacturer validation data for saliva were limited. Therefore, the salivary hormone concentrations in this study should be interpreted as standardized research measurements rather than directly interchangeable with serum-based clinical hormone values.

## 5. Conclusions

In conclusion, uncomplicated ongoing pregnancies were associated with higher salivary progesterone and estradiol levels and a higher P4/E2 ratio in early pregnancy. Lower salivary hormone levels and lower P4/E2 ratios were observed in women with progesterone-treated threatened miscarriage and in pregnancies ending in pregnancy loss. These findings support an association between saliva-based hormonal patterns and early pregnancy outcome, but they do not establish causality or independent prognostic value. Larger multicenter studies using adjusted predictive models and external validation are needed before these markers can be considered for clinical decision-making.

## Figures and Tables

**Table 1:** Baseline demographic and clinical characteristics of the study groups.

Variable	Uncomplicated Ongoing Pregnancies (*n* = 102) (1)	Threatened Miscarriage (*n* = 63) (2)	Miscarriage (*n* = 29) (3)	Statistical Analysis * Probability
Age (year)	23.0 [5.0]	28.0 [8.0]	22.0 [4.0]	χ^2^ = 25.760 *p* < 0.001 [2–1.3]
BMI (kg/m^2^)	25.0 [5.0]	26.0 [5.0]	24.0 [6.0]	χ^2^ = 8.400 *p* = 0.015 [1.2–3]
Employment status				χ^2^ = 2.137 *p* = 0.344
Yes	n 32% 31.7	n 19% 30.2	n 13% 44.8	
No	n 69% 68.3	n 44% 69.8	n 16% 55.2	
Education level				χ^2^ = 0.562 *p* = 0.967
Primary/middle school	29% 28.7	17% 27.0	9% 31.0	
High school	58% 57.4	36% 57.1	17% 58.6	
University	14% 13.9	10% 15.9	3% 10.4	
Income level				χ^2^ = 2.115 *p* = 0.715
Below minimum wage	38% 37.6	19% 30.2	10% 34.5	
Minimum wage	45% 44.6	29% 46.0	15% 51.7	
Above minimum wage	18% 17.8	15% 23.8	4% 13.8	

* “Pearson-χ^2^” cross tables were used to examine the relationships between two qualitative variables. For data not conforming to normal distribution, “Kruskal–Wallis H” test (χ^2^-table value) statistics were used to compare the measurement values of three or more independent groups, and values were presented as Median [IQR].

**Table 2:** Comparison of salivary progesterone, estradiol, and P4/E2 ratio at 6–7 and 8–9 weeks of gestation among the study groups.

Variable	Uncomplicated Ongoing Pregnancies (*n* = 102) (1)	Threatened Miscarriage (*n* = 63) (2)	Miscarriage (*n* = 29) (3)	Statistical Analysis * Probability
6–7 week				
Estradiol (pg/mL)	50.0 [26.5]	36.0 [28.0]	23.0 [5.5]	χ^2^ = 68.261 *p* < 0.001 [1–2.3] [2–3]
Progesterone (pg/mL)	1750.0 [800.0]	800.0 [400.0]	500.0 [100.0]	χ^2^ = 123.976 *p* < 0.001 [1–2.3] [2–3]
Progesterone-to-estradiol ratio	32.48 ± 3.94	22.80 ± 8.63	21.16 ± 1.89	F = 76.409 *p* < 0.001 [1–2.3]
8–9 week				
Estradiol (pg/mL)	95.0 [61.0]	61.0 [34.0]	29.0 [6.0]	χ^2^ = 94.066 *p* < 0.001 [1–2.3] [2–3]
Progesterone (pg/mL)	3400.0 [1600.0]	1400.0 [500.0]	700.0 [125.0]	χ^2^ = 139.582 *p* < 0.001 [1–2.3] [2–3]
Progesterone-to-estradiol ratio	33.22 ± 4.74	22.56 ± 8.06	21.77 ± 2.56	F = 84.425 *p* < 0.001 [1–2.3]

* For data conforming to normal distribution, “ANOVA” test (F-table value) statistics were used to compare the measurement values of three or more independent groups, and values were presented as Mean ± Standard deviation. For data not conforming to normal distribution, “Kruskal–Wallis H” test (χ^2^-table value) statistics were used to compare the measurement values of three or more independent groups, and values were presented as Median [IQR].

**Table 3:** Comparison of 6–7 and 8–9 week progesterone-to-estradiol ratios in the uncomplicated ongoing pregnancy group (*n* = 102) and the progesterone-treated threatened miscarriage with preserved viability group (*n* = 63).

Variable	Progesterone/Estradiol Ratio	Statistical Analysis * Probability
	6–7 weeks	8–9 weeks
Uncomplicated ongoing pregnancy (*n* = 102)	32.48 ± 3.94	33.22 ± 4.75
Progesterone-treated threatened miscarriage with preserved viability (*n* = 63)	22.81 ± 8.63	22.56 ± 8.06

* For data conforming to normal distribution, “Paired Sample” test (t-table value) statistics were used to compare the measurement values of two dependent groups, and values were presented as Mean ± Standard deviation.

**Table 4:** Comparison of salivary Estradiol results at 6–7 and 12 weeks of gestation in patients in the uncomplicated ongoing pregnancy group (*n* = 102) and threatened miscarriage group (*n* = 63).

Variable	Uncomplicated Ongoing Pregnancies (*n* = 102)	Threatened Miscarriage (*n* = 63)	Statistical Analysis * Probability
6–7 week Estradiol (pg/mL)	50.0 [26.5]	36.0 [28.0]	Z = −3.920 *p* < 0.001
12 week Estradiol (pg/mL)	184.0 [132.5]	61.0 [45.0]	Z = −9.025 *p* < 0.001

* For data not conforming to normal distribution, “Mann–Whitney U” test (Z-table value) statistics were used to compare the measurement values of two independent groups, and values were presented as Median [IQR].

## Data Availability

The original contributions presented in this study are included in the article. Further inquiries can be directed to the corresponding author.
